# Day-Case Total Hip Replacement for a Neck of Femur Fracture

**DOI:** 10.7759/cureus.83065

**Published:** 2025-04-27

**Authors:** Attar Singh, Harshadkumar D Rajgor, Thomas Moores, Mohmad Salim

**Affiliations:** 1 Orthopaedics, Walsall Manor Hospital, Walsall, GBR; 2 Trauma and Orthopaedics, Walsall Manor Hospital, Walsall, GBR; 3 Anaesthesia, Walsall Healthcare Trust, Walsall, GBR

**Keywords:** daycase surgery, neck of femur fracture, orthopaedics surgery, total hip arthroplasty: tha, total hip replacement (thr), trauma and orthopaedics

## Abstract

Total hip replacements are now commonly used in elective settings for patients in the NHS. With the rising costs associated with hospital admissions, there is an opportunity to apply day-case arthroplasty principles to total hip replacements in trauma patients with a neck of femur fracture. This report outlines a case of a patient in her sixties who presented with a fall and an intracapsular neck of femur fracture. She was admitted as an orthopaedic patient and treated with an emergency total hip replacement, given her good functional baseline and limited co-morbidities. Her health was optimised prior to surgery, and she successfully passed the necessary checks following surgery and was discharged from the hospital within 24 hours. By selecting the correct patients, optimising their health prior to surgery and following Getting It Right First Time (GIRFT) guidelines, it is possible to reduce the risk of complications, reduce the length of hospital stay and improve patient outcomes by favouring day-case total hip replacements in patients with neck of femur fractures.

## Introduction

Neck of femur fractures are one of the most common conditions which are admitted in orthopaedic departments across the UK, with an estimated cost of over £2 billion per year. They can be classified based on the location of the fracture, which in turn affects the management of the patient and the type of surgery. Intracapsular fractures are mostly managed with hemiarthroplasty due to the location of the fracture and the compromise to the blood supply to the femoral head [1]. The literature supports the recommendation for the long-term effectiveness of total hip replacements in acute neck of femur fractures, which can be implemented as a day-case procedure in the trauma setting for neck of femur fractures [2]. Currently, only patients who have a good functional baseline are considered for day-case total hip replacements, and in trauma settings, these patients are rarely seen. The current length of stay for a fractured neck of femur ranges from 15 to 30 days in the NHS [3].

## Case presentation

A 64-year-old female patient was admitted to Walsall Manor Hospital following a fall. She had sustained a mechanical fall onto her left hip. After the fall, she was unable to weight bear on her left leg. The patient denied any loss of consciousness, head injuries or any other injuries. She was otherwise well and denied having any other falls in the last year. Prior to the fall, the patient was able to mobilise independently without any walking aids, and her outdoor walking distance was over two miles.

The patient had a past medical history of ulcerative colitis, with a total colectomy, ileostomy and a rectal fistula in 2005, and asthma. She had no regular medications. The patient denied smoking and had a moderate alcohol intake (less than 10 units per week). With a Nottingham Hip Fracture Score of 0, the patient had a predicted 30-day mortality of 0.6% and AMTS of 10/10.

**Figure 1:**
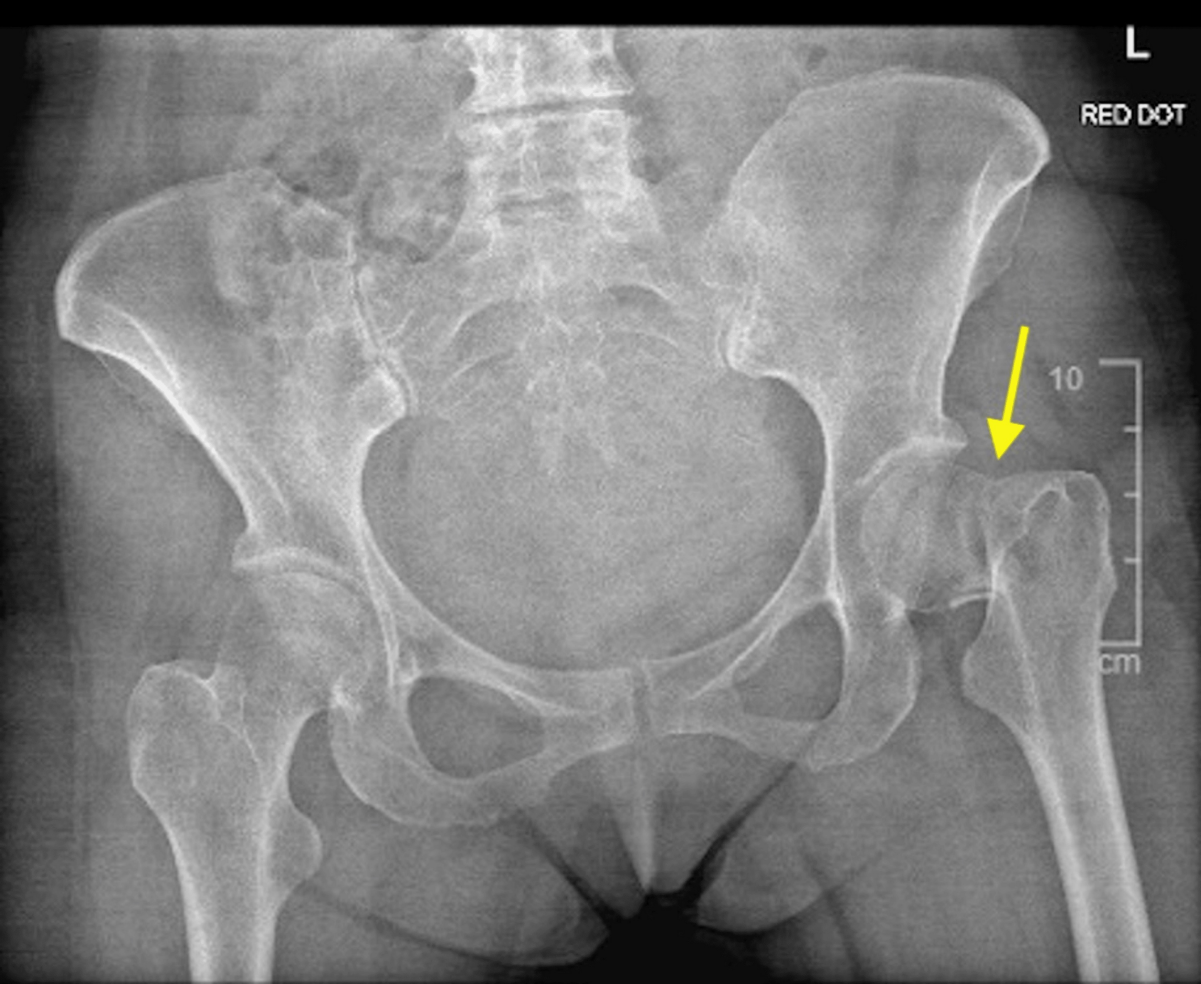
Pre-operative AP X-ray

**Figure 2:**
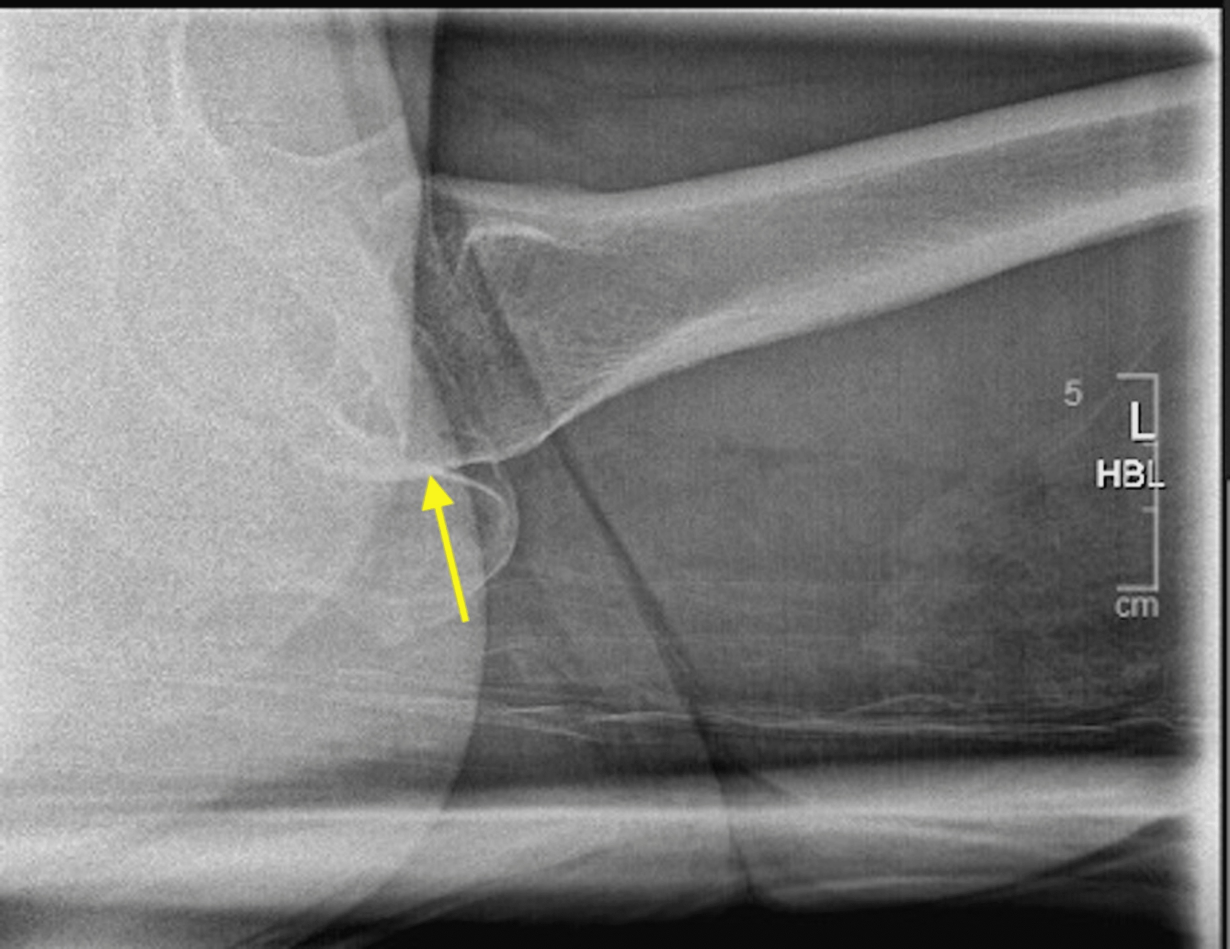
Pre-operative lateral X-ray

She had a pelvis X-ray, which confirmed an intracapsular neck of femur fracture, and was subsequently referred to the trauma and orthopaedics team (Figures 1, 2).

Pre-operative

Prior to being admitted to the ward, the patient had a fascia-iliaca block in the emergency department to provide more effective pain relief prior to the operation. She also had an ECG and chest X-ray to rule out any abnormalities that may affect her anaesthetic. Her blood tests were completed, which detected no significant abnormalities and two group and save samples were collected prior to the theatre.

The patient was marked and consented to a left total hip replacement. The risks and benefits of the operation were explained to her and given the patient’s good functional status, she was offered a total hip replacement as opposed to a hemiarthroplasty. The patient was also reviewed by the orthogeriatric consultant prior to surgery to optimise her health and improve the outcomes post-op.

Intra-operative

The patient was reviewed by the anaesthetic team and was deemed to be ASA (American Society of Anaesthesiologists) grade I. In the pre-operative brief, no concerns were raised. Therefore, the patient had a spinal anaesthetic, with tranexamic acid given at time of induction. She was brought into the theatre and WHO surgical safety checks were undertaken as per Trust guidelines. The patient underwent a left uncemented total hip replacement. 

There were no intraoperative concerns with good closure of the skin and infiltration of local anaesthetic. There was an estimated blood loss of 300-500 ml. Stability of the implant was tested in theatre, which showed excellent results. The patient was transferred to recovery and back to the ward with no further concerns.

Post-operative

**Figure 3:**
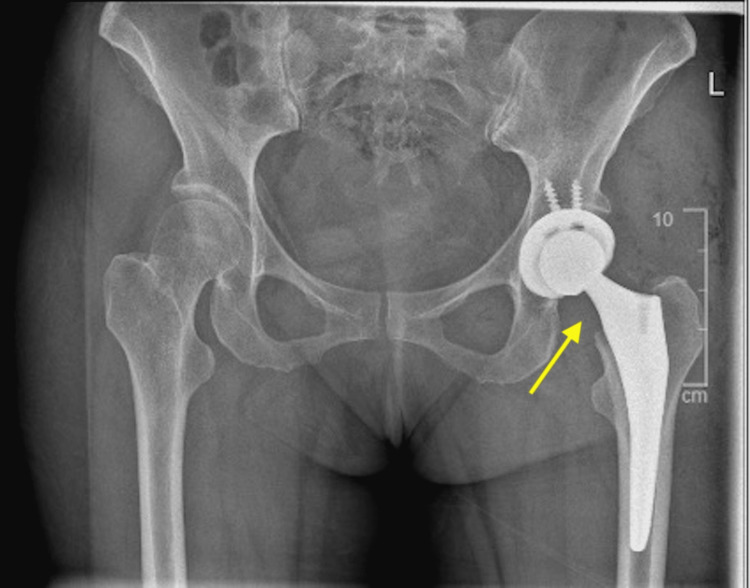
Post-operative AP X-ray

**Figure 4:**
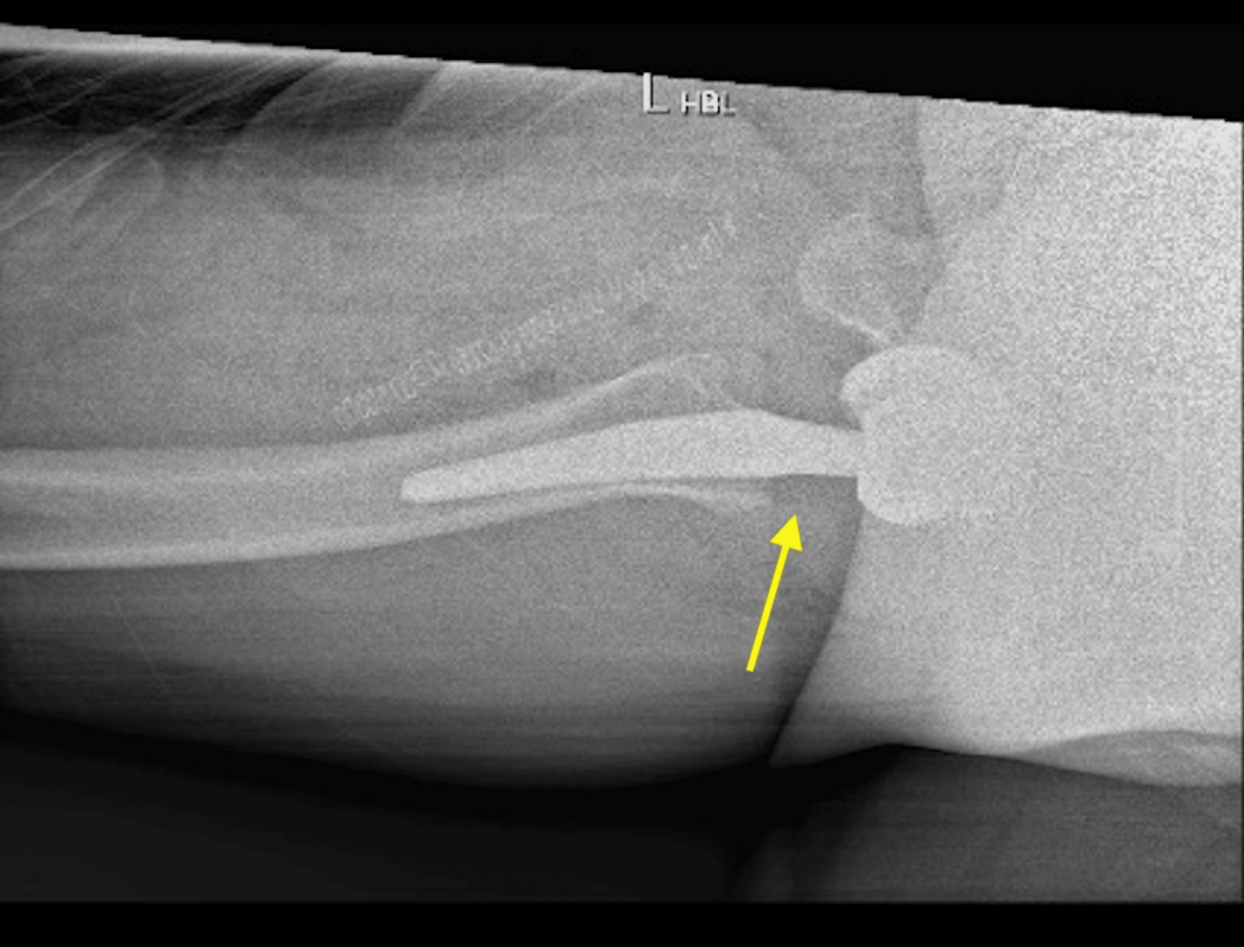
Post-operative lateral X-ray

We were able to follow standardised post-operative care procedures for this patient. Following return to the ward from recovery, she had an X-ray and a set of blood tests. The X-ray showed the prostheses in a satisfactory position with no sign of fracture or dislocation (Figures 3, 4). The blood tests also returned as unremarkable. In line with GIRFT (Get It Right First Time) guidelines, the patient was able to mobilise with physiotherapists on the same day of the operation, with the ability to perform ADLs (activities of daily living) independently including eating, dressing and walking. She also had a physiotherapy exercise plan to encourage mobilisation at home and a sturdy analgesic protocol to reduce the pain and encourage mobilisation in the first 48 hours post-surgery. Effective communication with the patient allowed for a safe discharge back home, including exercise and mobility advice, fall prevention information, wound care instructions and discharge booklets.

Follow-up

The patient was followed up at six weeks in the outpatient department. She was mobilising well since the operation with no signs of infection at the wound site. The patient was very pleased with the outcome of the surgery and was mobilising with one stick at the time of the appointment. She is due to be followed up again in three months’ time.

## Discussion

Total hip replacements are becoming increasingly prevalent as elective procedures within the NHS. As waiting times and bed costs continue to rise as well as the complications that can arise from prolonged hospital stays, it is ever more important to reduce the length of a patient’s hospital stay [4]. By applying the protocols of the enhanced recovery after surgery (ERAS), which were first implemented in colorectal surgery by Henrik Kehlet, it is possible to reduce the length of stay in hospital through measures which can be applied with the support of the multidisciplinary team [5]. Length of stay was shown to be significantly lower in elective patients in a district general hospital compared to trauma patients [6]. In turn, this leads to improved patient outcomes and satisfaction. It has also been shown that patients who have a day-case total hip replacement have reduced readmission rate at 30 days, which further demonstrates the importance of applying day-case procedure principles to trauma patients [7].

Whilst day-case procedures are not appropriate for all patients, there may be an opportunity to apply these principles in a trauma setting. With the principles of elective day-case total hip replacements in place, the advancement of anaesthetic technique, clinical technique, medication and physiotherapy, day-case arthroplasty may be able to improve outcomes for trauma patients [8]. Day-case total hip replacements have also been shown to improve health-related quality of life at one year for patients [9]. Using GIRFT principles, the elective hip replacement pathway can be applied to day-case trauma total hip replacements to ensure safe and effective management of the patient, and all of the relevant measures are put into place prior to discharge [10].

This case has shown how it is possible to apply elective day-case arthroplasty procedures to a trauma patient to facilitate faster discharge and reduced length of stay in the hospital. It does however rely on multiple factors which are often out of the control of the surgeon such as awaiting social care input, or availability of physiotherapists and occupational therapists. It has also been shown that there is a similar length of stay in total hip replacements as with hemiarthroplasties; therefore, it would indicate that if the patient is suitable, then a total hip replacement would be more beneficial [4].

The most significant factor associated with the relevance of day-case total hip replacements is understanding the past medical history of the patient. Often, those who have been admitted with neck of femur fractures will have multiple co-morbidities and/or have another reason for the admission, for example, a chest or urine infection. These are limiting factors that reduce the chance for a day-case total hip replacement to be undertaken. This often results in longer hospital stays due to optimisation or management of another medical condition, which can delay discharge. It is therefore key to ensure the inclusion criteria are suitable for the patient carefully applied to all patients to assess which patients are suitable for total hip replacements [11]. These can often include: no cognitive impairment, ASA grade 1 or 2, not a complex procedure, and independent of ADLs [12,13]. This also means that involvement of anaesthetists is required to determine ASA grade and develop an analgesia protocol to support discharge on the same day [8].

It is also important to understand the limitations of this case. We have shown that total hip replacements can be implemented in a trauma setting, however it has only been shown in one patient so far. On the other hand, there will be fewer patients who are admitted with neck of femur fractures who are suitable, but by identifying the correct patients, we can improve overall outcomes for patients. This means that we are also relying on the ASA grade of the patient as well as logistical measures in the hospital. For example, if an X-ray is unable to be arranged on the same day as the procedure, then it will lead to a stay overnight in the hospital, which in turn can increase the risk of hospital-related complications. We can also apply this when looking at discharge planning, taking blood tests, physiotherapy and analgesia protocols [4].

## Conclusions

In conclusion, we have been able to successfully implement day-case arthroplasty principles from the elective department to a trauma patient enabling a reduced length of stay, reduced complications and improved patient satisfaction. With a change in practices within trauma setting, there is a chance to reduce NHS costs and pressures within hospitals, as well as promote faster recovery and reduce complications related to extended hospital stays, including infections. In turn, there is the opportunity to research the effectiveness of day-case total hip replacements for trauma patients, with a view to improve logistical factors within the hospital.
